# Visible light may directly induce nuclear DNA damage triggering the death pathway in RGC-5 cells

**Published:** 2011-12-15

**Authors:** Guang-Yu Li, Bin Fan, Tong-Hui Ma

**Affiliations:** 1 Department of Ophthalmology, Second Hospital of JiLin University, ChangChun, China; 2 State Key Laboratory, Second Hospital of JiLin University, ChangChun, China

## Abstract

**Purpose:**

Visible light has been previously demonstrated to induce retinal ganglion cell (RGC)-5 cell death through the mitochondrial pathway. The present study was designed to determine whether visible light might also directly trigger the death pathway by damaging nuclear DNA.

**Methods:**

RGC-5 cells were exposed to various intensities and durations of visible light exposure. Cell viability and death were monitored with the 3-(4,5-dimethylthiazol-2-yl)-2,5-diphenyltetrazolium bromide assay and propidium iodide staining. Nuclear DNA damage caused by light was determined with the plasmid assay, genome DNA assay, and in situ terminal deoxynucleotidyl transferase dUTP nick end labeling. The subsequent activation of nuclear enzyme poly(ADP-ribose) polymerase-1 (PARP-1) was measured with western blot, and PARP-1’s role in the death pathway was assessed by using specific inhibitors. Poly (ADP-ribose) glycohydrolase and apoptosis-inducing factor (AIF) inhibitors were used to show their influence on light-induced cell death. Calcium influx was examined with the fura-2 assay and calcium channel blocker.

**Results:**

We found that visible light induced RGC-5 cell death in a time- and intensity-dependent manner. After the light intensity was increased to 2,600 lx, activation of the death pathway in RGC-5 cells was clearly observed by detecting double-strand DNA breaks and nuclear DNA damage in vitro. Nuclear enzyme PARP-1 was promptly activated after exposure to 2,600 lx of light for 2 days, and specific inhibitors of PARP-1 had significant neuroprotective effects. The poly(ADP-ribose) glycohydrolase inhibitor tannic acid and AIF inhibitor N-phenylmaleimide partially protected RGC-5 cells from light injury. A massive calcium influx was detected after 2 days of light exposure, and a calcium channel blocker partially protected cells against light injury.

**Conclusions:**

These results suggest that visible light exposure may directly cause nuclear DNA damage, which consequently activates PARP-1. In addition, RGC-5 cells damaged by 2,600 lx of light exposure can be used as an appropriate cell death model for screening neuroprotective drugs, since this treatment induced remarkable cell death within 2 days. Moreover, these results show that 2,600 lx of light exposure provides a more apparent activation of the death pathway than 1,000 lx of light exposure, which was used in a previous study.

## Introduction

The visible light wavelength ranges from 400 to 760 nm. Light with wavelengths below this range, such as ultraviolet (UV) and X-rays, is generally harmful to humans, and the majority of these waves is filtered out by Earth’s atmosphere. Wavelengths above this range are usually used in various communication and detection technologies, such as radio, radar, TV, and microwave. In the human eye, the cornea absorbs wavelengths below 295 nm, while the lens strongly absorbs wavelengths of light between 300 and 400 nm [1]. The cornea and the lens also absorb part of the infrared radiation wavelength range (980–1,430 nm), and the vitreous absorbs light at a wavelength above 1,400 nm [2,3]. Therefore, the wavelength of light that reaches the retina ranges between 400 and 760 nm.

Nevertheless, the same light that allows vision to occur is also potentially toxic to retinal cells in certain situations. The shorter wavelengths of light are known to interact with chromophores in photoreceptors as well as pigment epithelial cells and can cause oxidative stress and severe damage [4,5]. Indeed, the effects of short wavelength light are one cause of the loss of photoreceptor function in age-related macular degeneration [6,7]. However, recent studies have demonstrated that visible light can be a detrimental factor and induce retinal ganglion cell death, especially in cells where the function is already compromised, such as in glaucoma, diabetic retinopathy, and ischemia. Wood et al. [8] demonstrated that exposure to light was slightly, but significantly, harmful to healthy retinal ganglion cell (RGC)-5, a retina ganglion cell line, alone but was much more toxic to those cells undergoing serum deprivation. Retinal ganglion cell axons within the globe are functionally specialized by being rich with mitochondria, which produce the high energy required for nerve conduction and for maintaining optimal neuronal function. Osborne et al. [9] proposed that mitochondria could be the major target of visible light that leads to RGC injury. More recent evidence [10] has shown that visible light affects mitochondrial respiration and decreases mitochondrial homeostasis. In addition, our previous study demonstrated that the death pathway in RGC-5 cells induced by 1,000 lx of light exposure involved the activation of poly(ADP-ribose) polymerase-1 (PARP-1) and apoptosis-inducing factor (AIF) [11,12].

We believe that visible light not only influences mitochondria, which have been traditionally regarded as sensitive organelles in cells, but also affects the nucleus, which is an important center for DNA transcription and duplication. Here, we hypothesized that the nucleus of an RGC represents another organelle affected by light, and that light can directly cause DNA damage, which further activates the nuclear enzyme PARP-1. After increasing the light intensity to 2,600 lx, we found that visible light was able to directly induce double-stand DNA breaks and trigger downstream signals that ultimately lead to cell death. In addition, 2,600 lx of light exposure was found to be sufficient for the remarkable induction of cell death in RGC-5 cells within 2 days of treatment, which made the cell markers easier to detect. Therefore, this study provides a better cell death model for screening neuroprotective drugs.

## Methods

### Chemicals and reagents

Cell culture media and additives were obtained from Invitrogen (Beijing, China), and plastic cultureware was supplied by DingGuo BioTech (Beijing, China). The mouse anti-PARP-1 monoclonal antibody was purchased from BD Biosciences (No. 551024; Franklin Lakes, NJ). The mouse anti-β-actin monoclonal antibody (MAB1501) was obtained from Chemicon (Watford, UK), and the goat anti-AIF polyclonal antibody came from Santa Cruz Biotechnology (Santa Cruz, CA). Anticleaved caspase-3 and caspase-9 monoclonal antibodies were purchased from Cell Signaling Technology (Boston, MA). DNase I and biotin-16-dUTP were purchased from Boehringer Mannheim (Lewes, UK). Terminal deoxynucleotidyl transferase (TdT) was obtained from Promega (Southampton, UK), and the avidin-biotin complex kits were purchased from Vector Laboratories (Peterborough, UK). The antigoat IgG, the antimouse IgG, the antirabbit IgG, and all the other reagents and compounds were all purchased from Sigma-Aldrich (Shanghai, China).

### Cell culture

RGC-5 cells have been previously characterized and shown to express ganglion cell markers in culture [13]. RGC-5 cells, which were ordered from the American Type Culture Collection (ATCC, Manassas, VA), were grown in Dulbecco’s modified Eagle’s medium (DMEM; Invitrogen, Beijing, China) supplemented with 10% heat-inactivated fetal bovine serum, 100 U/ml of penicillin, and 100 mg/ml of streptomycin in a humidified atmosphere of 95% air and 5% CO_2_ at 37 °C. The doubling time of the cells was approximately 20 h under these conditions, and the cells were generally passaged by trypsinization at a ratio of 1:6 every 3–4 days.

### Light exposures

Light exposures of RGC-5 cells were performed as previously described [14]. Briefly, a normal culture incubator was equipped with two 8 Watt strip-lights that were completely covered with 2C UV filters that excluded light with a wavelength below 400 nm. The light source were placed about 20–60 cm directly over a tray with 6-, 24-, or 96-well plates in which the cells were growing. The intensity of the light directed onto the cells was determined by using a digital lux meter (LX-101; Lutron Electronic, London, UK). A paper box was placed in the same incubator to create a dark chamber used for the darkness control. DNA exposures were conducted in the same manner as the cells in the same settings. In this system, the cells maintained in the light or the dark were equally affected by any slight increase in the temperature of the incubator caused by the constant light source. The temperature of the media under the dark and light conditions was checked over a 1- to 3-day period, and no substantial differences were found.

### Cell viability assays and propidium iodide staining

Cell viability was assessed using a 3-(4,5-dimethylthiazol-2-yl)-2,5-diphenyltetrazolium bromide (MTT) reduction assay modified from that described by Mosmann et al. [15]. MTT was added to each well at a final concentration of 0.5 mg/ml in minimum essential medium (MEM) that lacked serum and phenol red and incubated for 1 h at 37 °C. Reduced MTT (blue formazan product) was solubilized with dimethyl sulfoxide, and the absorbance was determined using an automated microplate reader (Titertek Plus MS212; ICN Flow, Thame, UK) with a 570 nm test wavelength and a 690 nm reference wavelength. For propidium iodide (PI) staining, the cells were first cultured in a 24-well plate for 24 h. After the light exposure, the cells were then treated with the PI solution at a final concentration of 2 μg/ml and incubated in the dark for 10 min at room temperature. The PI-positive cells were scored on an inverted fluorescence microscope (Leica; Berlin, Germany).

### DNA preparation and electrophoresis

Genomic DNA from RGC-5 cells was extracted using a commercial kit (DV811A; Takara, DaLian, China) according to the manufacturer’s protocol or purified plasmid (PBR322; Takara) was dissolved in double distilled water. Each DNA extract was analyzed with a NanoDrop 1000 spectrophotometer (NanoDrop products, Wilmington, DE) to determine the DNA concentration using the A260/280 absorbance ratios. The purified genomic DNA or plasmid (PBR322) was exposed under light (2,600 lx) for 3 days in a 96-well plate. As the dark control, the same samples of equal concentration and amount were covered with a paper box placed in the same chamber. The genomic DNA or plasmid was finally analyzed and quantified with 1% agarose gel electrophoresis.

### Terminal deoxynucleotidyl transferase dUTP nick end labeling assay

A terminal deoxynucleotidyl transferase dUTP nick end labeling (TUNEL) assay was performed using the method previously described [16]. Cells grown on coverslips were fixed for 30 min with 4% paraformaldehyde in 0.1 M sodium phosphate buffer (pH 7.4), washed initially in Tris buffer (10 mM Tris-HCl, pH 8.0) for 5 min, and then exposed to 1% (v/v) H_2_O_2_ for 5 min to remove endogenous peroxidase activity. Following an additional wash in Tris buffer for 5 min, the cells were preincubated in TdT buffer (30 mM Tris-HCl, pH 7.2, 140 mM sodium cacodylate, and 1 mM cobalt chloride) for 10 min at 37 °C. The transferase reaction was subsequently performed by incubating the coverslips for 60 min at 37 °C with TdT buffer containing 0.25 units/ml of TdT and 40 mM biotin-16-dUTP. The reaction was stopped by incubating the coverslips in sodium citrate buffer (300 mM NaCl and 30 mM sodium citrate) for 2–15 min before blocking with 2% (w/v) BSA in PBS followed by a PBS wash. The coverslips were then incubated with avidin-biotin-peroxidase complex solution in PBS for 60 min at 37 °C. The labeled nuclei were visualized with 3,3′ diaminobenzidine (0.5 mg/ml) in 0.1 M sodium phosphate buffer (pH 7.4) containing 0.1% (v/v) H_2_O_2_. The cells on the coverslips were then washed in PBS before being mounted onto glass slides and visualized using a Zeiss microscope (Munich, Germany). The experiment was performed with three separate cultures with replicates of six coverslips per culture analyzed.

### Western blotting

RGC-5 cells were sonicated in protein lysate buffer (20 mm Tris-HCl, pH 7.4, 25 °C, 2 mm EDTA, 0.5 mm ethyl glycol tetraacetic acid [EGTA], 1 mm dithiothreitol, 50 mg/ml leupeptin, 50 mg/ml pepstatin A, 50 mg/ml aprotinin, and 0.1 mm phenylmethylsulfonyl fluoride). The bicinchoninic acid assay (BCA) method was used to estimate the protein level [17]. An equal amount (20 µg) of cell lysate was dissolved in sample buffer (62.5 mm Tris-HCl, pH 7.4, 4% sodium dodecyl sulfate, 10% glycerol, 10% β-mercaptoethanol, and 0.002% bromophenol blue), and the samples were boiled for 3 min. Electrophoresis was performed as previously reported [16] using 10% polyacrylamide gels containing 0.1% sodium dodecyl sulfate. Proteins were transferred to nitrocellulose membranes, and the blots were incubated for 3 h at room temperature with primary antibodies. The blots were then incubated with the appropriate biotinylated secondary antibodies. The blots were developed with a 0.016% w/v solution of 3-amino-9-ethylcarbazole in 50 mM sodium acetate (pH 5.0) containing 0.05% (v/v) Tween-20 and 0.03% (v/v) H_2_O_2_. The color reaction was stopped with a 0.05% sodium azide solution, and the blots were scanned at 800 dpi using an Epson Perfection 1200u scanner. Quantitative analysis of the files was performed using Labworks software (UVP Products; Upland, CA).

### Measurement of intracellular calcium concentration ([Ca^2+^]i)

[Ca**^2+^**]i in RGC-5 cells was measured using the fura-2 assay as previously described [18,19]. Briefly, cells on coverslips were loaded with 1.25 mol/l fura-2/AM by immersing the cells in Tyrode’s solution (100 mmol/l NaCl, 10 mmol/l KCl, 1.2 mmol/l NaH_2_PO_4_, 5 mmol/l MgSO_4_, 20 mmol/l glucose, 10 mmol/l taurine, and 10 mmol/l MOPS) containing Pluronic F-127 (0.025% w/v) for 30 min at 37 °C. The coverslips were then mounted into a chamber on the stage of an inverted fluorescence microscope (TE2000S; Nikon; Tokyo, Japan) with a xenon lamp and a CCD camera (Cool SNAP DG-4; Photomatrics, Tucson, AZ). Fura-2 emission (510 nm) was obtained by continuous rapid alternating excitation at 340 and 380 nm, and the 340/380 ratios were used to calculate [Ca**^2+^**]i. The [Ca**^2+^**]i standard calibration curve was obtained using the Fura-2 Calcium Imaging Calibration Kit (Invitrogen). Images were recorded and analyzed using MetaMorph/Fluor software (Molecular Devices Co., Sunnyvale, CA).

### Immunohistochemistry

Fixed cells were washed three times, and the cells were permeabilized and blocked for nonspecific epitopes with PBA buffer (1% BSA, 0.1% saponin, and 0.05% NaN_3_ in PBS) together with 2% donkey serum for 30 min at room temperature. Goat anti-AIF (Santa Cruz Biotechnology) primary antibody was incubated for 45 min in PBA at a 1:200 dilution. After the cells were washed three times with PBA, they were incubated for 30 min with an Alexa-labeled secondary Ab (A-11055; 1:1,000 dilution in PBA; Invitrogen). Nuclei were counterstained with 1 μg/ml 4′-6-diamidino-2-phenylindole. Cell fluorescence was detected and captured using a fluorescence microscope (DM RA; Leica).

## Results

### Light exposure decreases cell viability and induces cell death

**Figure 1:**
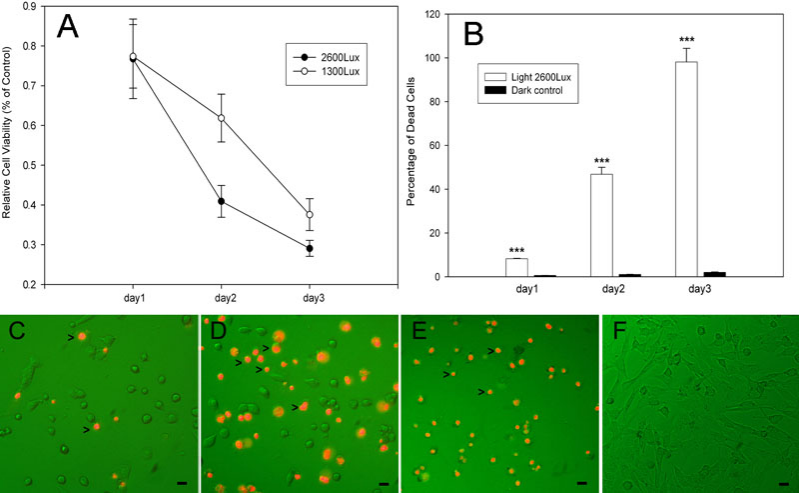
The exposure of visible light causes injury in retinal ganglion cell (RGC)-5 cells. **A**: The effect of visible light on the viability of RGC-5 cells over periods of 1–3 days was determined with the MTT assay. Results are expressed as a percentage of the control and are the means±SEM (n=48, three individual cultures). **B**: The percentage of dead cells was monitored from 1 to 3 days with PI staining. The results are the means±SEM (n=18, three individual cultures) (***p<0.001, one-way ANOVA and Bonferroni test). **C-F**: PI staining was used to show cell death. RGC-5 cells were exposed to 2,600 lx visible light for 1 day (**C**), 2 days (**D**), 3 days (**E**), or no light as a control (**F**). Dead cells were stained in red and are denoted with arrows (>). Scale bars=50 μm.

To measure cell damage caused by visible light exposure, cellular viability was first examined with the MTT assay. As shown in Figure 1A, the cell viability decreased to approximately 77.38% (1,300 lx) and 76.71% (2,600 lx) of that of the control cells after a 24 h exposure to visible light. On day 2, the cell viability decreased further to 61.86% (1,300 lx) and 40.91% (2,600 lx), respectively. However, on day 3, the cells showed the largest decrease in viability and had a remarkable decline to 37.55% (1,300 lx) and 29.04% (2,600 lx) viability compared to the control. Consistent with the reduction in cell viability, there was a pronounced increase in the percentage of cell death as detected with propidium iodide (PI) staining (Figure 1B-F). After a 24 h culture under the 2,600 lx light, the percentage of dead cells increased to 8.21%. Interestingly, substantial cell death occurred by day 2 (approximately 46.83%), and on day 3, cell death reached approximately 98.01%.

### Massive nuclear DNA breaks are generated after light exposure

**Figure 2:**
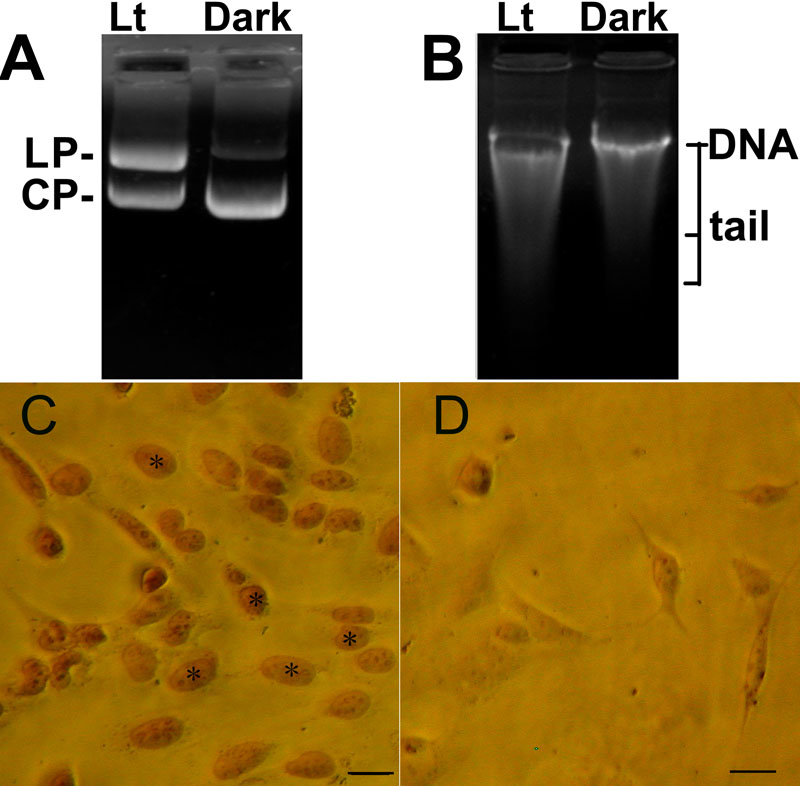
Light exposure induces DNA damage. **A**: The purified plasmid PBR322 was exposed to 2,600 lx light for 3 days and then examined with 1% agarose gel electrophoresis. LP, line plasmid. CP, circle plasmid. Lt, light situation. Dark, dark control. **B**: The isolated genomic DNA from RGC-5 cells was exposed to 2,600 lx light for 3 days and then examined with 1% agarose gel electrophoresis. Lt, light situation. Dark, dark control. **C, D**: The TUNEL assay was used to show the breakdown of DNA in dead cells. RGC-5 cells were exposed to 2,600 lx light for 2 days (**C**) or no light for 2 days (**D**). DNA strand breaks were positively detected with TUNEL and denoted with asterisks (*). Scale bars=15 μm.

We next assessed whether visible light was able to directly act on the nucleus and induce DNA double-strand breaks. To perform this assessment, we first used a purified plasmid to examine double-strand breaks induced by light exposure. As shown in Figure 2A, the fraction of linear plasmid above the circular plasmid band markedly increased after a 3-day light exposure (2,600 lx) as determined with agarose gel electrophoresis. Consistent with these data, the amount of the circular plasmid band decreased compared to the amount in the dark control group. We next examined whether visible light was able to directly cause genomic DNA fragmentation of isolated whole genomic DNA. Figure 2B shows that a 3-day light exposure significantly reduced the total amount of large DNA fragments (50 kb) but increased the degraded DNA fragments, which were identified by the relatively long tail (smeared band) with agarose gel electrophoresis. However, we did not detect these DNA changes with 1000 lx of light exposure in a previous study (data not shown). The light-induced DNA damage in situ was further confirmed with a TUNEL assay, which specifically labels DNA strand breaks and detects DNA fragmentation inside the cells. As shown in Figure 2C-D, several cells exhibited positive TUNEL staining of the nuclei after a 72 h light exposure, while staining was absent in the control cells. Thus, these results strongly suggest that visible light can directly cause DNA damage in RGC-5 cells.

### PARP-1 is activated in light-injured RGC-5 cells

**Figure 3:**
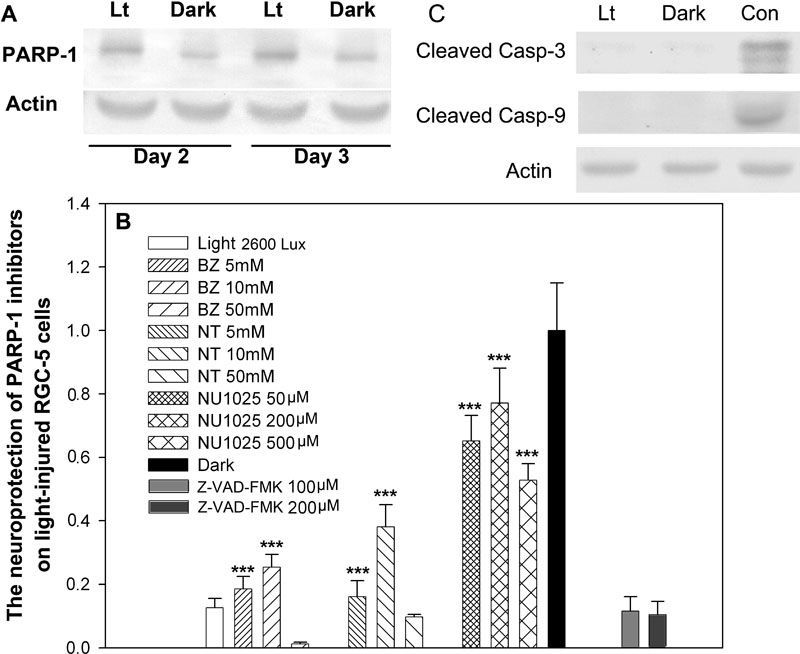
The PARP-1 inhibitors exhibit neuroprotection on light-damaged retinal ganglion cell (RGC)-5 cells. **A**: The upper panel shows representative western blots of PARP-1 and β-actin from cell lysates. **B**: The neuroprotection of light-damaged RGC-5 cells by PARP-1 inhibitors. RGC-5 cells were pretreated with PARP-1 inhibitors benzamide, nicotinamide, or NU1025 and the pan-caspase inhibitor Z-VAD-FMK and then exposed to 2,600 lx light for 3 days. Cell viability was determined with the MTT assay. Treatment with PARP-1 inhibitors significantly increased cell viability after exposure to light, while the pan-caspase inhibitor failed to protect RGC-5 cells from light injury. The results are expressed as a percentage of the control cells and are mean values±SEM (n=48, three separate cultures) (***p<0.001, one-way ANOVA and Bonferroni test). **C**: The active forms of cleaved caspase-3 and caspase-9 were not detectable in the light-treated cell lysates. Lt, light situation. Dark, dark control. Con, Etoposide-treated cell lysates as a positive control.

PARP-1 is one of the major nuclear enzymes responsible for repairing DNA strand breaks, but its over-activation usually causes energy depletion and induces cell death [20,21]. As shown by the western blot in Figure 3A, PARP-1 was remarkably upregulated after 2 days of light exposure compared to the control cells. Moreover, pretreatment with PARP-1 inhibitors (benzamide [22], nicotinamide [23], and NU1025 [24]) significantly increased the viability of light-injured cells, with approximately 25.4%, 38.1%, and 77.1% neuroprotection, respectively, relative to the control cells. However, the pan-caspase inhibitor, Z-VAD-FMK, failed to protect RGC-5 cells exposed to 2,600 lx light conditions, and we did not detect the active form of caspase-3 with western blot (Figure 3B,C). These results indicate that PARP-1 is a critical factor in the light-induced death pathway in RGC-5 cells.

### Poly (ADP-ribose) glycohydrolase and AIF inhibitors partially protect RGC-5 cells against light injury

**Figure 4:**
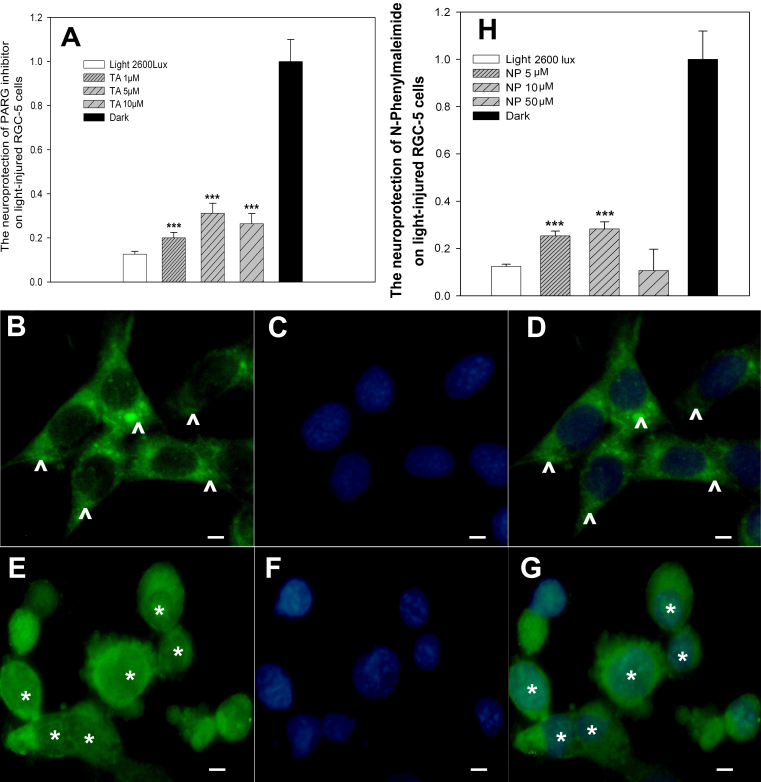
PARG and AIF inhibitors partially protect RGC-5 cells. **A**: The neuroprotective effect of a PARG inhibitor on light-damaged RGC-5 cells. RGC-5 cells were pretreated with the PARG inhibitor TA and then exposed to 2,600 lx light for 3 days. Cell viability was determined with the MTT assay. Treatment with TA significantly increased cell viability after light exposure. The results are expressed as a percentage of the control cells and are mean values±SEM (n=48, three separate cultures) (***p<0.001, one-way ANOVA and Bonferroni test). **B-G**: Following light exposure (2,600 lx, 3 days), some cells show AIF localization in the nucleus (*) while AIF in the control cells is primarily in the cytosol (arrows). **B**, **C**, **D**: dark control; **E**, **F**, **G**: light exposure. Scale bar=15 μm. **H**: Adding the AIF inhibitor NP (10 μM) to the culture medium attenuates the detrimental effect of light (2,600 lx for 3 days) on RGC-5 cells. Cell viability was measured with the MTT assay. Results are mean values±SEM for 3 different cultures. Each result was analyzed in quadruplet. Significant differences (***p<0.001) were determined with one-way ANOVA and Bonferroni tests.

Poly (ADP-ribose) glycohydrolase (PARG) may indirectly inhibit PARP activity by slowing the turnover of poly (ADP-ribose; PAR), limiting NAD^+^ depletion, and preventing the removal of PAR from PARP-1. We also found that treatment with tannic acid (TA) [25], a specific inhibitor of PARG, attenuated the cell death caused by light and increased cell viability by approximately 31.2% (Figure 4A). Since AIF is thought to be an important downstream factor in the pathway after PARP-1 activation and can induce large-scale DNA fragmentation [26,27], we next determined AIF localization after 2,600 lx of light exposure and AIF’s role in light-induced cell death.

Immunohistochemical studies showed that AIF localized to the nucleus after 3 days of exposure to 2,600 lx of light (Figure 4B-G). To evaluate the effect of AIF on light-induced cell death, an AIF inhibitor was used to determine whether blocking AIF could rescue RGC-5 cells from light damage. As shown in Figure 4H, pretreatment of the cells with 10 μM N-phenylmaleimide (NP) [28] increased cell viability from 15.72% to 29.35%, which demonstrated that AIF is involved in 2,600 lx light-induced cell death as well.

### Light exposure enhances calcium influx

**Figure 5:**
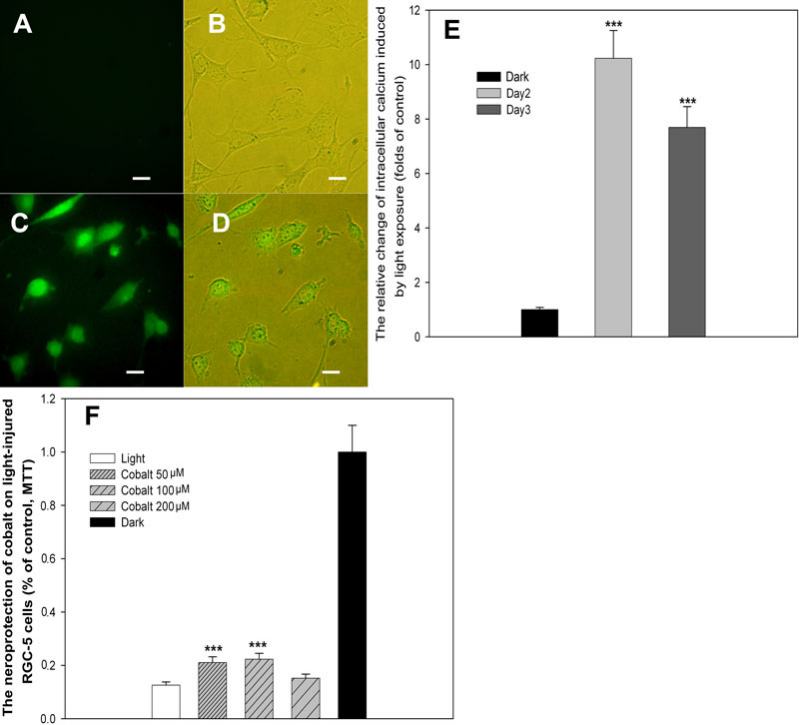
Light exposure causes massive calcium influx in RGC-5 cells. **A-D**: Calcium influx was detected with fura-2 with an inverted fluorescence microscope. **A, B**: Control; **C, D**: Cells treated with 2,600 lx light for 2 days; Scale bar=30 μm. **E**: Expanded overlay of typical responses was determined with calcium indicator. RGC-5 cells were cultured under the 2,600 lx light or with no light for 2 or 3 days. The intracellular free calcium concentration was measured using the fura-2/AM methodology. [Ca^2+^]i was significantly increased by light exposure, as indicated by the green fluorescence. **F**: RGC-5 cells were pre-cultured in normal media containing cobalt (50–200 μmol/l) and then exposed to 2,600 lx light for 3 days. Cell viability was determined with the MTT assay. Pre-treatment with 50–200 μmol/l cobalt significantly increased cell viability after light exposure. The results are expressed as a percentage of the control cells and are mean values±SEM (n=48, three separate cultures). (p<0.001, one-way ANOVA and Bonferroni test).

Intracellular calcium can be enhanced through calcium channel gating by ADP ribose, one of the products of poly(ADP-ribose) (PAR) [29]. We monitored the intracellular change of calcium after light exposure. As shown in Figure 5A-D, after a two-day light exposure (2,600 lx), there was a massive calcium influx into the cells, which was tracked with a fura-2 indicator. The maximal [Ca^2+^]i levels reached nearly 10-fold that of the control cells (Figure 5E). As a calcium channel blocker, cobalt [30] significantly increased cellular viability from 16.81% to 22.47%, as determined with the MTT assay (Figure 5F). However, we failed to detect calcium influx in RGC-5 cells exposed to 1,000 lx of light in a previous study (data not shown).

## Discussion

Krishnamoorthy et al. [13] were the first to derive the RGC-5 cell line by transforming post-natal day 1 rat retinal cells with the ψ2 E1A virus, and showed that the cells expressed thymus cell antigen 1(Thy-1), brain-3C (Brn-3C), Neuritin, *N*-methyl *D*-aspartate (NMDA) receptor, gamma-aminobutyric acid (GABA-B) receptor, and synaptophysin, but did not express glial fibrillary acidic protein (GFAP), syntaxin 1 and 8A1, a neurofilament marker (8A1). Another marker of retinal ganglion cells, γ-synuclein, is selectively and abundantly expressed in human RGCs in vivo and primary rat RGCs in vitro, and has also been detected in the immortalized RGC-5 cell line [31]. Moreover, RGC-5 cells possess several characteristics of progenitor neurons, such as being morphologically immature, dividing in culture, expressing nestin, and having the potential to differentiate into mature neurons [32]. RGC-5 cells can also be induced to differentiate by a nonspecific protein kinase inhibitor, such as staurosporine, which causes the cells to stop dividing and extend neurites that morphologically resemble mature neurons [33]. After differentiation, RGC-5 cells upregulate the ganglion cell marker Thy-1 and establish outward rectifying channels [34]. However, Van Bergen et al. [35] recently re-characterized RGC-5 cells, since they exhibit strong resistance to glutamate-induced excitotoxicity. Interestingly, the study showed that the cell line was from mouse (*Mus musculus*) and not rat (*Rattus norvegicus*) origin, based on mitochondrial and nuclear DNA analyses. By monitoring known markers of ganglion cells, the authors suggested that RGC-5 cells more likely represent a lineage of neuronal precursor cells [35]. Although the origin of this cell line is still controversial, it is still a potentially useful tool for studying neuronal pathophysiology and screening new neuroprotective drugs for treating nervous and visual system diseases.

The current study is the first to demonstrate a new mechanism for how visible light directly induces DNA damage in RGC-5 cells. Our results showing the light-induced increase in linear plasmid and fragmentation of purified genomic DNA indicated that visible light induced double-strand DNA breaks. In addition, the TUNEL assay showed that light exposure produced massive nuclear breaks in situ. The visible light exposure may directly induce photoproducts through the direct excitation of DNA, which is without a doubt critical for the double-strand DNA breaks and cytotoxic effects [36]. However, these data do not dispute the presence of other indirect mechanisms in cells. Osborne et al. [9] hypothesized that since retinal ganglion cells are laden with a large number of mitochondria that provide enough energy for the cells to conduct potentials, visible light might act on mitochondrial photosynthesizers, such as cyclooxygenase (COX), cytochrome P45 isoenzymes, and flavin protein to influence the electron transport system and generate substantial reactive oxygen species (ROS). The ROS, including peroxynitrite anion (ONOO)-, are able to penetrate the nuclear membrane and cause various oxidative DNA modifications, such as 8-hydroxyguanine (8-oxoG*), DNA strand breaks, and sites of base loss [37–39]. These two possible pathways may work together to cause nuclear DNA damage, which then further triggers downstream signals and induces cell death.

PARP-1 is a well conserved nuclear enzyme that repairs DNA damage and is extremely sensitive to various DNA lesions. In addition, PARP-1 activation occurs much earlier and exceeds the magnitude of the augmentation of DNA nicks, as monitored with a TUNEL assay [40]. Upon binding to breaks in DNA, PARP activity is increased as much as 500 fold [41,42] and catalyzes the transfer and polymerization of ADP-ribose units onto PARP and other nuclear proteins, including histones and DNA topoisomerases I and II [43,44]. In our experiments, we found that PARP-1 was remarkably upregulated after 2,600 lx light exposure, which indicated that massive nuclear DNA damage had occurred in the RGC-5 cells.

The role of PARP in DNA repair suggests that PARP activation serves to help rescue damaged cells. Although this can occur following mild DNA damage, the opposite is true in cases of excessive DNA damage. Activation of PARP in response to massive DNA damage induces rapid and drastic alterations in the metabolic pools and pathways. Moreover, PARP-1’s DNA repair activity is saturated by a finite level of DNA damage and then decreases with additional DNA damage. Massive levels of DNA damage elicit strong activation of PARP, which leads to cell death from NAD and ATP depletion [45,46]. In the present study, after testing three different PARP inhibitors, we found that PARP-1 plays a critical role in light-induced death in RGC-5 cells. Blocking PARP-1 provided significant neuroprotection against light damage. Benzamide analogs, including benzamide itself and nicotinamide, are first-generation inhibitors, which are competitive with the substrate NAD^+^ and interfere with binding to the active site of the enzyme [23]. In our light experiments, we found that 10 mM of benzamide or nicotinamide increased cell viability to 25.4% and 38.1%, respectively, compared to the control groups. Since first-generation inhibitors have several disadvantages, such as low potency, a short cellular residence time, and nonspecific interactions, we tested new-generation inhibitors as well, including phthalazine-1(2H)-one inhibitor NU1025 and quinazolinone AZD2281 (Olaparib; AstraZeneca, London, UK). In light-damaged RGC-5 cells, NU1025 exhibited excellent neuroprotection and increased the cell viability to 77.1% compared to the control, which almost completely reversed the cell death caused by light. In addition, NU1025 has an IC_50_ in the low micromolar range [23].

PARP-1 activation uses a massive amount of cellular NAD^+^ to form PAR [47]. However, PAR has a fast turnover rate, with a half-life of approximately 1 min due to rapid degradation by the endo-exoglycosidase PARG [48,49]. Presumably, blocking PARG may indirectly inhibit PARP activity by slowing the turnover of PAR, limiting NAD^+^ depletion, and preventing the removal of PAR from PARP-1. To further understand the role of PARP-1 in the light-induced RGC-5 cell death pathway, we used an MTT assay to examine the neuroprotection of a PARG inhibitor. The results showed that the PARG inhibitor TA [25,50] markedly reduced the cell death of RGC-5 cells under light exposure.

apoptosis inducing factor (AIF), a downstream factor of PARP-1 activation, is translocated to the nucleus after exposure to 2,600 lx of light. AIF is a mitochondrial enzyme anchored in the inner membrane of the mitochondrial space during normal physiologic conditions. Upon the induction of cell death, the mitochondrial form of AIF is cleaved to yield a soluble proapoptotic protein that translocates from the mitochondria to the cytosol and nucleus where the AIF interacts with DNA and/or RNA to cause caspase-independent chromatin condensation and large-scale DNA fragmentation [26,27]. In our experiments, we found that N-phenylmaleimide (NP), a specific inhibitor of AIF, was able to partially block cell death and increase cell viability from 15.72% to 29.35% after light exposure compared to the control cells. These results indicate that AIF is activated during 2,600 lx light exposure and is involved in the light-induced death pathway.

Calcium (Ca^2+^) is an important and universal intracellular second messenger, which modulates many cell functions, including susceptibility to cell death [51]. In light-induced cell death, we detected Ca^2+^ influx in the RGC-5 cells using a fura-2 indicator. Three days of light exposure boosted the intracellular Ca^2+^ concentrations by nearly 10-fold compared to the control group. In addition, 200 µM of the Ca^2+^ channel blocker cobalt was able to partially attenuate cell death. Light can induce the over-activation of PARP-1, which then generates massive PAR; however, intracellular calcium can be enhanced through calcium channel gating by ADP ribose, one of the products of PAR degradation [29]. High intracellular Ca^2+^ accumulation alters the permeability of the mitochondrial membrane, which inhibits mitochondrial ATP production and promotes necrosis [52]. In addition, Ca^2+^ also activates the calmodulin-regulated enzyme nitric oxide synthase to produce large amounts of nitric oxide (NO) [53], which combines with superoxide to form the much more reactive peroxynitrite an ion (OONO^−^). Peroxynitrite damages the nuclear membrane and leads to oxidative injuries of DNA, resulting in robust PARP activation and the subsequent activation of PARP-dependent cell death [54–56].

To our knowledge, this study is the first demonstration that visible light exposure may directly cause nuclear DNA damage, which in turn activates PARP-1 to trigger death in RGC-5 cells. In addition, we found that RGC-5 cells damaged with 2,600 lx of light may be a more appropriate cell death model, since most of the RGC-5 cells died within 3 days of treatment and markers of the death pathway were more clearly detected than in cells treated with 1,000 lx of light. These findings will greatly speed up our future research and will save a substantial amount of time in screening neuroprotective drugs.
